# Application of Smartphone Technologies in Disease Monitoring: A Systematic Review

**DOI:** 10.3390/healthcare9070889

**Published:** 2021-07-14

**Authors:** Jeban Chandir Moses, Sasan Adibi, Sheikh Mohammed Shariful Islam, Nilmini Wickramasinghe, Lemai Nguyen

**Affiliations:** 1 School of Information Technology, Deakin University, 1 Gheringhap St, Geelong, VIC 3220, Australia; jcmoses@deakin.edu.au; 2 Institute for Physical Activity and Nutrition (IPAN), Deakin University, Burwood, VIC 3125, Australia; shariful.islam@deakin.edu.au; 3 Iverson Health Innovation Research Institute, Swinburne University of Technology, Hawthorn, VIC 3122, Australia; nwickramasinghe@swin.edu.au; 4 Department of Information Systems and Business Analytics, Deakin Business School, 221 Burwood Highway, Burwood, VIC 3125, Australia; lemai.nguyen@deakin.edu.au

**Keywords:** technology, smartphone applications, wearable sensors, disease monitoring, mobile solutions, disease management, technology acceptance, chronic disease, COVID-19, patient-generated health data

## Abstract

Technologies play an essential role in monitoring, managing, and self-management of chronic diseases. Since chronic patients rely on life-long healthcare systems and the current COVID-19 pandemic has placed limits on hospital care, there is a need to explore disease monitoring and management technologies and examine their acceptance by chronic patients. We systematically examined the use of smartphone applications (apps) in chronic disease monitoring and management in databases, namely, Medline, Web of Science, Embase, and Proquest, published from 2010 to 2020. Results showed that app-based weight management programs had a significant effect on healthy eating and physical activity (*p* = 0.002), eating behaviours (*p* < 0.001) and dietary intake pattern (*p* < 0.001), decreased mean body weight (*p* = 0.008), mean Body Mass Index (BMI) (*p* = 0.002) and mean waist circumference (*p* < 0.001). App intervention assisted in decreasing the stress levels (paired *t*-test = 3.18; *p* < 0.05). Among cancer patients, we observed a high acceptance of technology (76%) and a moderately positive correlation between non-invasive electronic monitoring data and questionnaire (r = 0.6, *p* < 0.0001). We found a significant relationship between app use and standard clinical evaluation and high acceptance of the use of apps to monitor the disease. Our findings provide insights into critical issues, including technology acceptance along with regulatory guidelines to be considered when designing, developing, and deploying smartphone solutions targeted for chronic patients.

## 1. Introduction

Globally, human life expectancy has increased considerably over the years and continues to rise [1,2]. This improvement in life expectancy can be attributed primarily to the remarkable advancement in healthcare services, medical procedures, diagnostic technologies, improved living conditions, health literacy, awareness about nutrition, and other public health measures [2]. It is predicted that by 2050, the number of children under 14 years will be outnumbered by the elderly population aged over 65 years, mainly due to increased life expectancy and decreased fertility rate [1]. However, with this rise in life expectancy coupled with general advances in healthcare, we are simultaneously witnessing a rise in chronic conditions such as diabetes and cardiovascular diseases [3]. Furthermore, it is estimated that around 15% of the world’s population is living with some form of disability [4,5]. Overall, in 2016, the major causes of death (54%) worldwide were due to non-communicable diseases (e.g., ischaemic heart disease, stroke, chronic obstructive pulmonary disease, Alzheimer disease and other dementias, cancers (trachea, bronchus, and lung), and diabetes mellitus) [6].

Advancements in knowledge, science, and technology have been critical in curbing the global mortality rate and prolonging life expectancy [7]. Moreover, the progress in data science, such as big data analytics and cloud technologies, has enabled the development of personal life expectancy prediction models, which could be used to calculate health indexes from massive datasets collected over the years [8]. In recent times, the widespread application of Information and Communication Technologies (ICTs) in the health sector has resulted in significant improvements in the healthcare delivery system, such as promoting patient-centred healthcare, improving quality of care, and educating health professionals and patients [9]. ICTs, including digital technologies for electronic capture, storage, processing, and information exchange, have been used in chronic disease prevention and management [9,10], and are used in chronic disease monitoring and surveillance such as to track cancer cell progression (e.g., SO_2_ metabolism) [11], predict the risk of diabetic complications (e.g., foot ulcer) [12], and remote monitoring and training of stroke and Parkinson’s patients [13]. ICTs are also used as self-management tools to assist patients with diabetes [14], cancer [15], and dementia [16]. Moreover, with the constant growth and evolution of ICTs, a vast amount of information related to patient care is generated; hence there is a need for decision support systems to provide knowledge, models, and data processing tools in assisting medical practitioners to take appropriate decisions when treating patients [17]. Nevertheless, the slow progression and subtle nature of some chronic disease symptoms question the capacity of ICT monitoring devices to detect the acute episodes of these conditions in real-time [18].

Smartphone technology, including apps [19] and app integrated wearable sensors [20], offers further chronic disease management potential. Moreover, the technological capacity, popularity, availability, and increased smartphone ownership globally, including in developing nations, promotes the smartphone as an attractive tool to assist patient self-management, continuous symptoms and vital sign monitoring, and communication between patients and physicians [21,22]. Furthermore, chronic disease management apps are beneficial in improving patients’ clinical outcomes and assisting in providing necessary medical care [22]. Additionally, apps integrated with built-in smartphone sensors and wearable external sensors can capture numerous health parameters to deliver personalised healthcare solutions [23,24]. Because apps have the potential to capture data using the various smartphone sensors, they are used for various forms of health monitoring, including heart, eye, skin, mental health and activity monitoring, respectively, implying a vital need for regulatory policies for the development of smartphone-based healthcare systems [25]. 

Patients with chronic conditions are often dependent on life-long healthcare systems that involve different stakeholders, including healthcare professionals, specialised care centres, primary care providers, and community-based services [26,27]. However, the inconsistent implementation of quarantine policies due to COVID-19 has limited access to hospital care for patients with chronic diseases, created uncertainty over their disease status, and accentuating their emotional situation [28]. Furthermore, COVID-19 highlights the imperative need for countries to ensure an equitable and accessible healthcare system to meet people’s emerging health needs, such as people-centred, affordable, non-discrimination and equitable care, tailored to the individual’s needs [29].

The literature contains reviews on the effectiveness of technology such as smartphone applications (app), and wearable sensors, for chronic disease management. A recent systematic review found that technology could facilitate adherence to treatment of chronic diseases using simple messages and alerts [30]. However, another systematic review observed a paucity of data on the effectiveness and use of mobile and web-based apps that support self-management and transition in adolescents with chronic diseases [31]. Other reviews specifically looked at the effectiveness of wearable sensors in adults with a chronic cardiometabolic disease [32] and chronic kindly disease [33]. We are unaware of any recent literature review which has evaluated the use of app integrated smartphone technology in monitoring and managing chronic disease in adult populations, irrespective of their medical condition. Hence, this review aims to evaluate the accuracy and technology acceptance of app integrated smartphone technologies with standard clinical procedures among adults living with chronic conditions.

## 2. Materials and Methods

We followed Preferred Reporting Items for Systematic Reviews and Meta-Analyses (PRISMA) guidelines to organise the review [34]. Since this review is based on peer-reviewed studies for which primary investigators obtained informed consent, ethics approval was unnecessary [35].

### 2.1. Protocol

This review is undertaken due to the unprecedented situation which has arisen due to COVID-19 and hence it was not pre-registered in PROSPERO.

### 2.2. Data Sources and Search Strategy

The studies were identified through a comprehensive literature search in the online database, including Medline complete, Web of Science, Embase, and Proquest. Initial literature search focused on technologies for disease monitoring and management using a combination of key search terms which include ‘technology OR computer OR tablet OR mobile phone OR smartphone OR internet OR app’, AND ‘disease OR illness OR sickness OR condition OR disorder OR health’, AND ‘monitoring OR tracking OR evaluation OR tool’. We included studies published in English. Although several apps are developed and deployed for chronic disease management [36,37,38], there is a paucity of evidence for the effectiveness and use of mobile and Web-based apps in chronic disease management [31,39]. Consequently, with the overwhelming numbers and types of digital solution, the World Health Organisation (WHO) has recommended their evaluation of in research settings to rigorously analyse the benefits, harms, acceptability, feasibility, resource use and equity considerations of digital health interventions before deployment [39]. Hence, we have considered studies evaluated in research settings and published exclusively in research journals. The final searches were based on the Embase search strategy (Table S1), and searches were adapted as appropriate to the specifications of the databases [40].

### 2.3. Study Selection Criteria

A set of selection criteria were used to narrow down the selection of articles that would fulfil the main objectives of the systematic review (Table 1). The search is limited to a timeframe between January 2010 and September 2020 matching the period of disruptive innovations in wireless technologies such as Bluetooth, ZigBee, and 4G, and app development for health applications (mHealth) [38,41,42].

### 2.4. Study Selection Process

**Figure 1:**
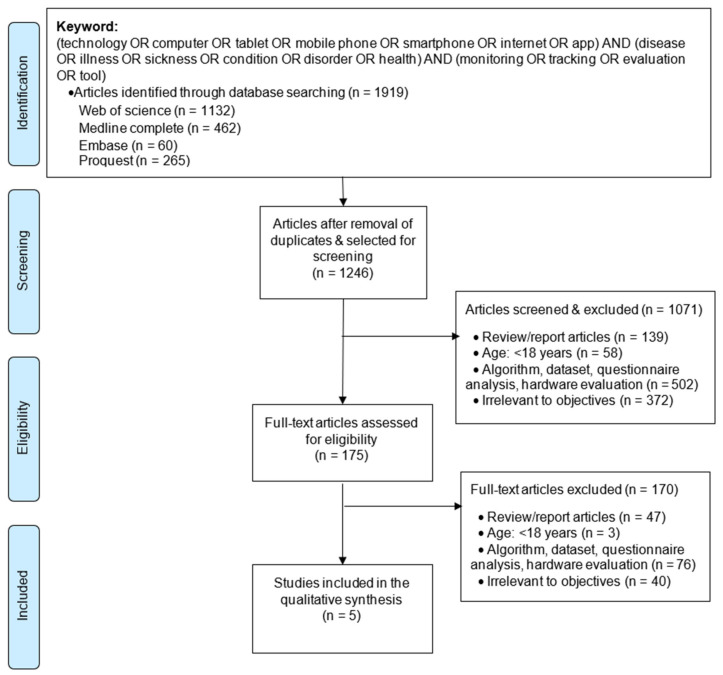
Flow diagram for selection of articles; (adapted from reference [34]).

We followed a step-by-step selection process to identify the relevant articles (Figure 1). The obtained citations were imported into the reference management software EndNote and duplicates removed. We applied the selection criteria to screen the articles and to select studies relevant to the objective of this review.

### 2.5. Data Extraction

One reviewer independently evaluated the titles and abstracts of all records identified in the initial database search [43]. The reviewer then assessed the full-text for eligibility in line with the inclusion criteria [43]. Data were extracted regarding the app and integrated wearable devices, the questionnaire used, and monitoring duration. The outcomes of each study were considered. The acceptance of technology by the various stakeholders was also extracted. Additionally, study characteristics, including study type and participant types, were also extracted.

### 2.6. Risk-of-Bias Assessment

The studies considered in this review had used an app to monitor and manage the chronic condition. We performed risk assessment at the outcome level based on heterogeneity and availability of data [34]. Two dimensions define risk, considering the probability of an event occurring that could lead to harm and the severity of the harm likely to follow that event [45]. Moreover, due to the lack of a clinically relevant risk assessment framework for medical apps [45], we have used the Two-dimensional “App-space” for risk assessment of mobile medical apps as an appropriate risk assessment tool. The Two-dimensional “App-space” categorises types of risk variable as inherent to the app, including specific risk variables such as intended functions, inaccurate or out of date content, the complexity of the task supported by the app, and lack of feedback or failsafe mechanism, and as external factors depending on the context of app use including specific risk variables such as app user, inappropriate app usage, inadequate user training, likelihood of errors being detected, and app usage factor [45]. Nevertheless, increases in risk with increase in app functionality and a risk score corresponding to the magnitude of this risk are assigned for each factor [45]. For example, apps used for BMI calculations are at negligible risk compared to apps used as clinical decision support tools, which are at high risk [45]. 

### 2.7. Analysis of Selected Studies

Primary outcomes for the research articles and their statistically significant value where applicable are represented. This has enabled a critical review of each study’s results and identification of consistencies in their functional capability and differences in applicability. The studies have considered that the critically significant cut-off point of the results, as designated by *p*-values, were at the level of α < 0.05.

## 3. Results

### 3.1. Search Results

We retrieved a total of 1919 articles for the applied search key from the database. Upon removing duplicates, there were 1246 articles for full-text review. Further, we screened the electronically obtained titles and abstracts for relevance of selection criteria. The selected 175 articles were further accessed, assessed, and matched with the study’s objectives. At each level of article screening, we excluded review articles such as systematic reviews [46], surveys [47], literature reviews [48], protocols [49] and similar articles. We further screened for non-human subjects such as battery health monitoring [50], structural health monitoring [51], pipe structural health monitoring [52], and similar articles, including the use of health information technology to calculate mortality risks [53]. Additionally, since we focused on chronic disease monitoring and management, we discarded studies focusing on evaluation and development of research tools, such as on assessing the quality of health apps [54], gauging the effectiveness of apps using a paper-and-pencil survey [55], evaluating patient attitudes toward mobile phone-based health monitoring [56], development of prototypes not evaluated on humans [57], and other such studies. Likewise, we eliminated studies focused on the economic evaluation of health technologies [58,59], undertaken amongst children, i.e., aged below 18 [60,61,62], and undertaken among less than fifteen participants [63]. Finally, we included five papers in this review and Table 2 represents the characteristics of the included studies. Table S2 represents the summary of findings.

### 3.2. Search Results

The app is used as the primary source to capture data [64,65,66,67,68], in addition to wearable sensors [64,67] and questionnaires [64,65,66]. The sample size of the studies varied between 16 [68] and over 900 [65]. The studies used variable lengths of time to monitor the interventions in home settings such as eight weeks [65], twelve weeks [64,66], and 24 weeks [68], whereas a single session observation/monitoring to evaluate the accuracy of wearable smart clothing system for cardiac health monitoring was conducted in the clinical setting [67]. The studies included both experimental [65,66,67] and observational [64,68] types, and were conducted in multiple international locations including two studies in Asia [66,67], three studies in Europe [64,68,69], and a study in the United States of America [65].

### 3.3. Risk Assessment

The risk associated with the medical apps at app level and external (contextual) level is evaluated (Table 3) [45]. At app level, the risk of apps used to monitor terminal cancer patients [64], and heart failure (HF) patients [67] remotely, and to regulate the stress of borderline personality disorder (BPD), were at high risk. This high risk is due to any programming error which could cause inherently dangerous medical functionalities resulting in an associated risk to the patient [45]. Moreover, since the studies had not detailed the features of apps [64,65,66,67,68], we could not access the risk associated with the app content and feature such as availability of feedback mechanism [45].

The risk associated with external factors was minimal for the app user and there was little inappropriate app usage in all the considered studies, since the app was used in research settings by the intended users. However, the risk associated with other factors varied. For example, although all the studies had recruited proficient smartphone participants and demonstrated the system’s functionalities [64,65,66,67,68], the users lacked the knowledge to discern the accuracy of the content delivered through the app, increasing the associated risk [65,68]. Likewise, the risk of detecting errors is high in the app used amongst terminally ill cancer patients with a life expectancy of less than a year, due to the criticality and necessity of accurate data capture [64]. Furthermore, the study undertaken to evaluate an app’s effectiveness for eating disorder management is available in the app store to be downloaded by the public, having a very high app usage factor and increasing risk, since a faulty app could affect comparatively many users [65]. 

The risk is high in apps used for disease monitoring including for monitoring of cancer patients in palliative care [64], cardiac health monitoring of HF patients [67], and monitoring and reducing aversive tension in BPD patients [68]. In contrast, a lower risk is involved in using apps for disease management, including the treatment of eating disorder [65] and weight management [66].

### 3.4. Disease Monitoring and Management

**Figure 2:**
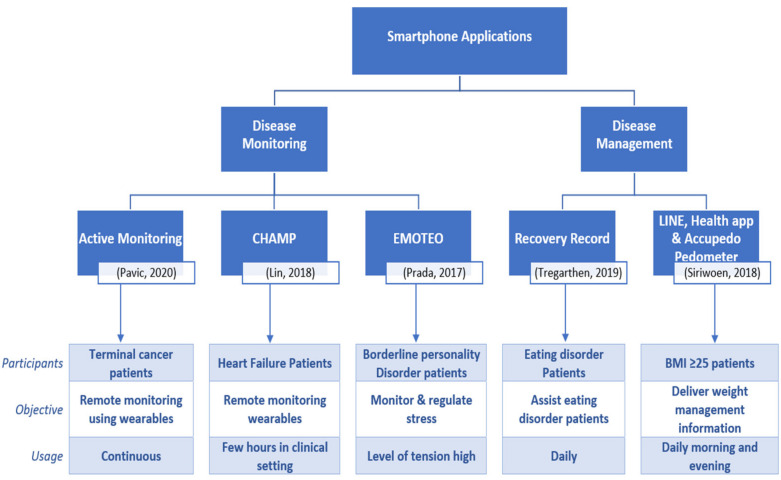
App functionalities and classification.

Disease is an interruption, cessation, or disorder of body functions, systems, or organs [70]. The cause of disease varies; pathogens, which are invading agents, cause infectious disease, whereas unhealthy lifestyle choices, environmental factors, and genetic disorders can cause non-infectious disease [70]. Although chronic disease may be less severe, frequent/continuous medical attention is needed to overcome health impairments limiting daily living activities [70]. Screening for chronic disease could prevent onset [71]. However, after onset, there is an imperative need to manage and monitor the disease [72,73]. Disease monitoring involves the continuous collection of data to assess the status of health and disease conditions [73], whereas the disease management approach equips the individual patient with the information and skills necessary to act as their own self-managers, thereby maintaining optimal health [72]. Accordingly, we have classified the studies into disease monitoring [64,67,68] and disease management [65,66] depending on the objectives and functionalities of the study. Figure 2 represents the classification and functionalities of the apps, and participant characteristics.

#### 3.4.1. Disease Monitoring

The feasibility of remote monitoring using an app integrated wearable sensor (bracelet worn on the upper arm) was assessed among terminal cancer patients who had an estimated survival period of between 8 weeks and 12 months to anticipate and prevent the rapid deterioration of health conditions, eventually minimising unplanned readmissions or emergency hospital visits [64]. In addition to the sensor captured data including resting heart rate, resting heart rate variability, and speed of steps, the participants filled-in daily the app-based Visual Analog Scale (VAS) questionnaire for pain and distress, and on analysing the responses a moderately positive correlation between mobile health features extracted from sensor signals and daily VAS ratings (r = 0.6, *p* < 0.0001) was found [64]. On the contrary, participants were interviewed over the phone weekly to record response for the European Organization for Research and Treatment of Cancer Quality of Life Questionnaire-Core 30 (EORTC QLQ-C30) and no significant correlation between mobile health features and the individual QLQ-C30 scores was found [64]. Furthermore, patients who had an emergency hospital visit (*n* = 11, 36.7%) during the study period exhibited an increased resting heart rate, decreased heart rate variability, and a trend towards increased step speed when compared with patients not having an emergency hospital visit [64].

To provide early risk warnings, i.e., to predict the left ventricular ejection fraction (LVEF) among HF patients, a multi-channel mechano-cardiogram (MCG) wearable smart clothing system for cardiac health monitoring integrated with hardware, firmware, app, and wireless design features was designed and evaluated in clinical settings [67]. Although the study duration was for a short time, an accuracy rate of up to 96% for predicting cardiac functions’ abnormality, such as HFs, was observed [67].

There is an essential need to regulate inner tension of BPD sufferers since they could exhibit adverse self-harming behaviours ranging from self-cutting to burning and banging the head against the wall, at any time of the day [68]. Corresponding to the current aversive tension level recorded in the app by the participants, the app suggested random exercises between 3 and 8 min, played in audio/video format for the patient to complete [68]. There was a decline in the level of stress (Initial: (5.95 ± 3.13) points: (Final: 2.83 ± 2.36) points (paired *t*-test = 3.18; *p* < 0.05)), signifying that apps can be efficient in reducing aversive tension [68]. 

#### 3.4.2. Disease Management

The potential of apps in managing eating disorders, which can severely impact psychological, physical, and social functioning amongst participants (*n* = 959) categorised into a standard group (*n* = 458) and intervention group (*n* = 501), was evaluated [65]. The standard group participants used the standard app, which functions via a cognitive behavioural therapy concept [65]. In contrast, the intervention group received a tailored version of the app that included algorithmically determined clinical content, explicitly aligned to the user [65]. The self-reported Eating Disorder Examination Questionnaire [EDE-Q] score determined the magnitude of the eating disorder in each participant, and at week four from study commencement participants in the intervention group (51.5%; 227/441) as well as in the standard group 46.2% (156/338) achieved a clinically meaningful change in EDE-Q score, i.e., a decrease in the EDE-Q global score by a 0.5 SD [65]. Furthermore, at week eight, 61.6% of intervention group participants (180/292) and 55.4% of standard group participants (158/285) achieved a clinically meaningful EDE-Q score [65]. However, the rate of remission on the EDE-Q at eight weeks was significantly greater in the intervention group (d = 0.22; *p* ≤ 0.001) when compared with the standard group [65].

Participants who were overweight and obese but willing to act on weight loss participated in a 12-week weight management program delivered through a collection of apps [66]. Apps with diverse functionalities such as counting daily steps walked and messaging applications for providing food and beverage, along with easy exercises as multimedia content and photos of the food consumed, were collectively used to assist the participants in weight loss [66]. The study observed a significant decline in the mean weight and waist circumference of the participants (*p* = 0.008, *p* < 0.001) from baseline (72.2 ± 10.4 kg, 92.1± 10.1 cm) during post-intervention (week 6: 71.6 ± 10.8 kg, 89.9 ± 9.9 cm) and follow-up (week 12: 71.4 ± 11.0 kg, 87.8 ± 10.7 cm) periods, respectively [66]. Furthermore, providing weekly tailored interventions could favourably influence behavioural factors such as healthy eating and physical activity (*p* = 0.002), eating behaviours (*p* < 0.001), dietary intake pattern (*p* < 0.001), mean body weight (*p* = 0.008), mean BMI (*p* = 0.002), and mean waist circumference (*p* < 0.001) [66]. Additionally, other lifestyle changes such as decreased consumption of sugar-sweetened beverages (*p* < 0.001) and increased frequency of taking stairs (*p* = 0.002) could have influenced the positive outcome in weight management [66]. Furthermore, a marked increase in the rate of obesity was observed among participants aged 30 years and older, with the highest rate being in those between 45 and 59 years of age (51.8%) [66].

### 3.5. Technology Acceptance

There is a need to perform usability testing in a realistic scenario and environment to evaluate ease of use and usefulness of the solution and to determine system acceptability [74]. Accordingly, studies have used various methods such as customised questionnaires [64], duration of app usage [68], and the Technology Acceptance Model (TAM) [67] to evaluate technology acceptance (Table 4). The majority (76%) of the terminal cancer patients using wearable sensors assessed for usability of app and comfort of the wearable sensor in a real-life scenario in a 12 weeks study period had a positive experience with the monitoring system [64]. In contrast, a study considered the extended time duration of the app usage, such as the number of sessions per week (11.95 ± 9.75), frequency of recordings per day (1.21 ± 1.12), and overall time exposed to the device (318.1 ± 166.7 min) per subject, with a mean session of around 2.73 (±4.43) minutes as the indicator for user satisfaction [68]. Furthermore, a TAM-based path analysis confirmed perceived ubiquity and perceived benefits to be key determinants of user acceptance of wearable sensors [67]. The participants in this study show a positive user attitude toward of using wearable sensors.

### 3.6. Regulatory Agencies

The revolution in smartphone technologies, direct-to-consumer genetic testing, crowd-sourced information, and big data have enabled researchers, including independent researchers, citizen scientists, patient-directed researchers, do-it-yourself (DIY) researchers, and self-experimenters, in facilitating the development of mHealth systems [75]. On the other hand, the easy access to mHealth systems increases the potential of unregulated health research [75], which could be beneficial but could also pose risks to the users involving accuracy, privacy, and safety [76]. Moreover, there is an ambiguity regarding when a medical app could be considered as a formal medical device [45]. Hence there is an imperative need for governments to regulate the development and deployment of mHealth systems through competent regulatory agencies [75,76]. 

The considered studies had used apps to remotely monitor patients through wearable sensors [64,67], and to assist in the participant’s health and wellbeing [65,66,68]. According to Two-dimensional “App-space” for risk assessment of mobile medical apps, it is mandated that apps that assist in clinical decision support should undergo formal assessment and regulation by a professional and government body such as the Food and Drug Administration (FDA) and Medicines and Healthcare Products Regulatory Agency (MHRA) [45]. Nevertheless, disease monitoring apps including for monitoring of cancer patients in palliative care [64], cardiac health monitoring of HF patients [67], and monitoring and reducing aversive tension in BPD patients [68], have not sought approval for use by professional and government bodies. However, they could pose a significant risk to patients due to a combination of inherent complexity, functionality, and potential to cause health hazards if misused [45]. Furthermore, apps assisting in functionalities, including diagnostic support, patient decision making, and medical calculators, are subjected to formal assessment by local health organisations [45]. Accordingly, studies evaluating disease management, including in treating eating disorders [65] and weight management [66], have sought local health organisation approval.

## 4. Discussion

The studies displayed a skewed representation of male [64,67] and female [65] participants. A study to evaluate the app’s effectiveness in weight loss [66] and to regulate the stress of BPD patients [68] did not consider male participants but found the intervention to be effective. However, there is a void in understanding the interventions’ impact amongst male participants [66,68]. Moreover, the exploration of gender-based intervention outcomes could help conceptualise the role of gender in addressing its relationship with intervention outcomes [77]. 

The increase in the complexity of app functionality proportionately increases the possibility of harm due to usage [45]. Although the apps used were for critical healthcare needs, including disease monitoring [64,67,68] and disease management [65,66], no formal assessment and regulation by professional and government bodies were undertaken. Currently, there are efforts to formulate guidelines in prescribing apps and outline key issues to enable app dissemination in healthcare [78]. However, since apps are developed and shared more quickly than they can be assessed for efficacy, safety, and security, an unwarranted situation to for patients and clinicians in which the challenge of distinguishing helpful from harmful apps is created [79]. Additionally, currently, there are a lack of clear and accepted standards for the development (planning, requirement analysis and research, design, and application testing) of healthcare apps which could pose different risks to developers, providers, patients and the public [80]. Consequently, there is a necessity to formulate guidelines for the development of apps to mitigate risks, including clinical, privacy, and economic risks [80].

The advancement in smartphone technology and the ability to integrate with various adjunct technologies has enabled apps to be developed and deployed in health and clinical practices [81]. Studies used apps which were freely available [65,66,68], and had been developed with a potential to be integrated with wearable sensors to monitor targeted activities [64,67]. Apps used in home settings have assisted in the improvement of health and wellbeing of the participants [65,66,68], and were found to be feasible in remotely monitoring patient’s vital organs using integrated wearable sensors [64]. Furthermore, although an app to assist in eating disorders is freely available globally via the Google Play (Android) and iTunes (iPhone) app stores, the study considered participants from a selected geographical location and observed that underserved individuals with eating disorder symptoms might benefit clinically from a self-help app [65]. These observations suggest that apps could play a vital role in achieving clinically meaningful improvement in patients, irrespective of the economic status of the country, due to cost-effectiveness and the ability to reach more individuals [65].

Cancer was the second leading cause of death globally in 2018 [82]. A study found that it is feasible to remotely monitor terminally ill cancer patients with an app-integrated wearable sensor system [64]. Nevertheless, the detection of cancer at an early stage could drastically reduce the risk [83]. However, screening faces hindrance due to barriers such as insufficient knowledge about cancer, the purpose of screening [84], emotional aspects [85], and a variety of personal, practitioner, test-related and logistical factors [86]. Furthermore, the restrictions enforced in hospital visits globally due to COVID-19 have forced cancer screening programmes to halt, risking the chances of early diagnosis [87]. In contrast, the use of smartphone-based telemedicine has enabled palliative care physicians to follow-up on cancer patients during the COVID-19 pandemic period and enabling symptom management, restocking of opioid medications, and providing information regarding oncological treatments requiring consultation with other departments [88]. 

Smartphone-based telemedicine has good prospects in the future of healthcare delivery systems amongst cancer patients, especially among those unable to visit hospitals regularly due to a weakened immune system [88]. There is a scope for smartphone integrated technologies in biomedical image analysis including detection for skin/breast cancer [83,89,90], cancer pain management [91], treatment of breast cancer [92], and dissemination of information to help cancer patients in non-clinical settings [93]. Although recently several apps have been developed for cancer management, a study revealed that only 3% of apps (*n* = 4/123) had been evaluated by healthcare providers [94]. However, the advancement in smartphone technology creates an opportunity to capture continuous real-time data benefitting cancer management [95]. Hence, there is a need for multidisciplinary collaboration to develop mobile sensing frameworks that could deliver timely and personalised support to patients through remote monitoring of their health in order to improve clinical oncology outcomes [95].

COVID-19, a respiratory illness caused by a highly contagious virus with the potential for fatal outcomes to specific risk groups, is currently a global public health concern [96]. Given the high infectivity rate of the virus, the growing demand for telehealth and the monitoring of infected patients are on the rise [97]. A study observed that the risk factors for weight gain during self-quarantine are inadequate sleep, snacking after dinner, lack of dietary restraint, eating in response to stress, and reduced physical activity [98]. This study observed that apps could effectively alleviate mental stress and assist in eating disorder and weight management [65,66,68]. Hence, there is scope for apps and integrated technologies to function as useful tools to maintain individual’s health and wellbeing during this pandemic period. Furthermore, it is premature to precisely predict the physical, psychological, neuropsychological and social impacts of COVID-19 [99]. The study observed that app-integrated wearable sensors have the potential to remotely monitor vital readings, irrespective of the severity of the disease and of age [64,67]. Furthermore, a technologically dependent monitoring system could have the potential for remote and continued monitoring and management of patients with/recovered from COVID-19 to assist healthcare practitioners [100]. Hence, this unprecedented situation calls for technology [101] and a multidisciplinary research approach to monitor recovered patients [102].

Technology acceptance is evaluated using different methodologies [64,67,68], though a few studies have excluded this [65,66]. The primary concern expressed by around 50% of terminally-ill cancer patients regarding withdrawal from the study were due to the handling of devices being too cumbersome [64]. Moreover, studies have recruited participants who were proficient smartphone users [64,65,66,68]. Therefore, while developing a healthcare system with various ICT components, it must be secure, reliable, socially acceptable, and above all be usable by various stakeholders, i.e., the system must support different user groups to interact effectively and easily, and also assist older people in accessing the system effortlessly [74]. Furthermore, there is an imperative need to evaluate the system using established models such as TAM to examine the significance of the outcomes in a global setting.

### 4.1. Implications for Practice

The reviewed articles suggest that apps could be used to monitor and manage chronic diseases with accuracy on par with that of the current gold standards [64,65,66,67,68]. Furthermore, in conjunction with apps, wearable sensors are also used to capture real-time recordings of vital signs of patients and have assisted in predicting health and wellbeing [64,67]. In the current situation, apart from phone calls, the usage of apps and wearable sensors could assist in making well-informed decisions to maintain health [97,103].

There are clinical, privacy, and economic risks involved with the use of healthcare apps [80]. Furthermore, there is a lack of clear and accepted standards for the development of healthcare apps which could pose different risks to developers, providers, patients and the public [80]. Moreover, there is ambiguity regarding when a medical app can be considered as a formal medical device [45]. Hence, there is an imperative need for governments to regulate the development and deployment of mHealth systems through professional and/or government agencies [75,76].

### 4.2. Implications for Research

The findings of this research have implications for future technology design and development. Despite multiple health apps being available to support the health and wellbeing of individuals, there is a paucity in knowledge of app credibility. For example, are the readings verified against clinical standards, are the devices medically conformed, is the transferred information secure and private, and was the app evaluated in a research study and among different populations [37,38]? Our findings would be of assistance to health practitioners for the design and deployment smartphone technologies in clinical practice. For example, apps integrated with wearable sensors to monitor targeted activities amongst terminal cancer patients and HF patients can monitor vital signs continuously in real-time irrespective of the disease severity and participants age [64,67]. Developing smartphone integrated wearable sensors could help health practitioners monitor their chronic patients continuously, in real-time, but remotely. Likewise, this finding would also help individuals in self-managing behavioural factors such as physical activity and eating habits using apps with research evaluation, which could help them stay physically and mentally fit. For example, apps could effectively alleviate mental stress and assist in eating disorder and weight management [65,66,68]. Hence, individuals could benefit from apps evaluated in research settings to self-manage their health. Moreover, there are research prospects in undertaking systematic literature reviews considering factors such as development, approval from regulatory agencies, evaluation in research settings and other factors, to recommend apps as healthcare assistive tools during the pandemic, and in developing countries lacking infrastructure.

#### Future Research Directions

Healthcare practitioners prescribe apps as a useful self-management tool for patients [104]. This review highlighted that apps could be used to monitor and manage chronic diseases with accuracy on a par with that of the current gold standards [64,65,66,67,68]. With advancements in technology, big data analytics can improve outcomes for patients in providing evidence-based healthcare solutions [105,106]. It is anticipated that health parameters recorded from the smartphone may increase the prospect of analysing vital recordings to offer well-informed personalised healthcare solutions [107]. Moreover, the application of machine learning, which is a branch of artificial intelligence using a large amount of digital data, could extract new knowledge that could help healthcare practitioners make well-informed decisions [108]. However, while developing ICT based healthcare systems, in addition to the requirements of the stakeholders, the privacy of users and security of the data must be considered [108]. Furthermore, it is foreseen that mobile technologies, data analytics, Internet of Things (IoT), and 5G connectivity could enhance the healthcare system by providing evidence-based, personalised healthcare solutions that are capable of handling increased velocity, variety, and volume of healthcare data with privacy and security in real-time [107]. Hence, there are research prospects for the design, development and deployment of integrated healthcare systems with mobile technologies, data analytics, and IoT.

Diabetes patients constituted one-third of COVID-19 infected cases, and mortality was high and was independently associated with glycaemic control and body mass index (BMI) in addition to other complications, including CVD and renal complications [109]. Moreover, diabetes independently increases the adverse impacts of COVID-19 with modifiable factors (e.g., HbA1c), having a significant but modest impact compared with comparatively static factors (e.g., race and insurance), indicating an urgent and continued need to mitigate severe COVID-19 risk in this community [110]. With COVID-19 as an on-going crisis, the risk is high among diabetes patients of facing health deterioration due to poor lifestyle and risk of mortality if infected with COVID-19. Developing integrated smartphone systems to monitor and manage diabetes patients is a current global need and would assist diabetes patients, their family and caregivers, and their health practitioners.

Symptom severity associated with COVID-19 ranges from a mild common cold-like illness to severe viral pneumonia leading to acute respiratory distress syndrome that is potentially fatal; complications related to severe COVID-19 also include, but are not limited to, multi-organ failure, septic shock, and blood clots [99]. Although mHealth provides several benefits to healthcare practitioners due to its sophisticated tools, only reliable and valid solutions should be included in medical practice [111]. Despite the many health apps available, there is a paucity of knowledge regarding app credibility [37,38]. Hence, a literature search to discover potential health consequences due to the virus infection, changes and restrictions in lifestyle, health and wellbeing, social gathering and disease screening, monitoring, and managing technologies currently available would be of assistance to the healthcare community and all adults globally, including COVID-19 patients.

### 4.3. Limitations of This Survey

The review reports only on a small part of the disease prevention process, although technology has found widespread application in disease prevention and monitoring. We have exclusively considered articles published in research journals, following the WHO guidelines in selecting studies undertaken in research settings. However, this has resulted in excluding grey literature and could have incurred publication bias. Although studies have observed that it is feasible to monitor and manage disease using app and integrated technologies [64,65,66,67,68], the results should be considered cautiously due to limitations such as small sample size [64,66,67,68], absence of consideration of gender [66,68], and being conducted in a single geographical location [64,65,66,67,68]. Furthermore, one reviewer performed article screening and data extraction, which could present a possibility of bias [43]. The heterogeneity of the data and the lack of standard models such as TAM to evaluate the user perception of the information system have also restricted us in conducting this meta-analysis. However, these studies’ findings could be extended to a large sample size, including male and female participants, to validate the findings’ accuracy.

## 5. Conclusions

We found a significant relationship between app use and standard clinical evaluation in disease monitoring and management. Furthermore, app integrated disease monitoring technologies have received high acceptance amongst patients. Although the apps used were for critical healthcare needs, including disease monitoring and management, no formal assessment and regulation by professional and government bodies was undertaken. Hence, our findings provide insights into critical issues, including technology acceptance and regulatory guidelines that must be considered when designing, developing, and deploying smartphone solutions targeted at chronic patients.

## Figures and Tables

**Table 1:** Selection criteria for review articles (adapted from reference [44]).

**Inclusion Criteria:**
Data collected through electronic tools: e.g., smartphone applications and wearable sensors. Human subjects living with chronic disease. Participants aged ≥18 years. Number of participants ≥15. Year of publication: January 2010–July 2020. Examine the use of technology and disease monitoring and management.
**Exclusion Criteria:**
Publications on incomplete or part of research (e.g., editorials, abstracts, workshop/conference summaries, research proposals, descriptive survey, clinical protocols, research methods, literature reviews, conceptual papers). Participants aged <18 years. Non-human focused (e.g., building, physical structures, bridges, health economic, evaluation of study ethics). Use of non-electronic tools to collect data (e.g., paper-based questionnaire, opinions, viewpoints). Evaluation and development of research tools (e.g., hardware and algorithm improvement studies, and clinical measurement technology to access and analyse secondary data).

**Table 2:** Characteristics of included studies.

Articles	Study Type	Country	Count	Age	Male (%)	Female (%)	App	Wearable	Questionnaire	Clinical Evaluation	Duration (Weeks)
(Pavic, 2020) [64]	Obs.	Swiss	30	64	71	29	Active monitoring	Bracelet	Yes	-	12
(Tregarthen, 2019) [65]	Exp.	US	959	µ:34	6	94	Recovery record	-	Yes	-	8
(Siriwoen, 2018) [66]	Exp.	Thailand	38	25–52	-	100	LINE, Health app, Accupedo Pedometer.	-	Yes	Yes	12
(Lin, 2018) [67]	Exp.	Taiwan	48	20–90+	77	23	CHAMP	Smart vest	Yes	Yes	Few hours in clinic.
(Prada, 2017) [68]	Obs.	Swiss	16	18–50	-	100	EMOTEO	-	-	-	24

Obs: Observation, Exp: Experimental, App: Smartphone application, Swiss: Switzerland, US: United Nations.

**Table 3:** Risk assessment of selected studies.

Articles		(Pavic, 2020) [64]	(Tregarthen, 2019) [65]	(Siriwoen, 2018) [66]	(Lin, 2018) [67]	(Prada, 2017) [68]
Inherent to the app.	Intended function	High-risk	Low risk	Low risk	High-risk	High-risk
	Inaccurate or out of date content	Not specified				
	Complexity of task supported by the app	High-risk	Low risk	Low risk	High-risk	High-risk
	Lack of feedback or failsafe mechanism	Not specified				
External factors, depending on context of app use.	App user	Yes	Yes	Yes	Yes	Yes
	Inappropriate app usage	No	No	No	No	No
	Inadequate user training	Low risk	High-risk	Low risk	Low risk	High-risk
	Likelihood of errors being detected	High-risk	Low risk	Low risk	Low risk	Low risk
	App usage factor (AUF)	Low	High	Low	Low	Low

**Table 4:** Technology acceptance evaluation.

Article	Patient Medical Condition	Technology Acceptance Evaluation Methodology	Response	Comments
(Pavic, 2020) [64]	Terminal cancer.	Customised questionnaire	76% appreciated the monitoring. 93% felt the wearable comfy. Bracelet-worn on 53% study days. Smartphone used on 85% study days.	Remote monitoring of palliative cancer patients using app integrated wearables is feasible.
(Tregarthen, 2019) [65]	Eating disorder.	Not Specified		
(Siriwoen, 2018) [66]	BMI ≥25.			
(Lin, 2018) [67]	Heart failure.	Technology Acceptance Model	Positive response in 8/10 criteria.	Remote cardiac monitoring using wearables is feasible.
(Prada, 2017) [68]	Borderline personality disorder.	Duration of app usage	(11.95 ± 9.75) sessions per week. (1.21 ± 1.12) recordings per day. (318.1 ± 166.7 min) per subject overall time exposed to the app.	Decrease in aversion tension. Higher app usage statistics is proportion to the effectiveness of the app.

## Data Availability

Source data for all figure(s) and number(s) are provided with the paper and Supplementary Materials.

## Supplementary Materials

The following are available online at https://www.mdpi.com/article/10.3390/healthcare9070889/s1. Table S1: Embase search strategy; Table S2: Study objectives and outcomes.
