# Supplementary material for: Application of Smartphone Technologies in Disease Monitoring: A Systematic Review
Source: Healthcare (Basel). 2021 Jul 14;9(7):889. doi: 10.3390/healthcare9070889 (PMC8303662; doi:10.3390/healthcare9070889)
Supplement: Supplementary file 1 [file healthcare-09-00889-s001.zip › healthcare-1247872-supplementary.pdf]

Table S1. Embase search strategy.

#	Searches	Results
**1**	technology OR computer OR tablet OR mobile phone OR smartphone OR internet OR app	**180,175**
**2**	disease OR illness OR sickness OR condition OR disorder OR health	**2,206,213**
**3**	monitoring OR tracking OR evaluation OR tool	**890,260**
**4**	1 AND 2 AND 3	**1022**
**5**	Limit 4 to January 2010 to September 2020 publication duration	**690**
**6**	Limit 5 to controlled clinical trial and randomized controlled trial	**60**

Table S2. Study objectives and outcomes.

Articles	Objective	Result(s)	Outcome(s)
Pavic, 2020 [44]	To monitor vital signs.	• A moderate positive correlation (r=0.6, *p*<0.0001) between health features extracted from sensor signals and daily VAS ratings.<br>• No significant correlation between mobile health features and individual QLQ-C30 scores.	• 76% positive response for continuous health monitoring using wearables.<br>• The monitoring of palliative cancer patients using wearables is feasible.<br>• Mobile health features might be promising biomarkers to predict unplanned hospital readmissions.
Tregarthen, 2019 [45]	To assist in healthy eating.	• EDE-Q outcome improvement: Tailored group (61.6%) & Standard group (55.4%).<br>• Tailored group: > rate of remission on the EDE-Q at 8 weeks (d=0.22; *p*≤0.001).	• Individuals with eating disorder symptoms may benefit clinically from a self-help app.<br>• Personalised self-help may be more effective in promoting symptomatic remission than a generic app.
Siriwoen, 2018 [46]	To assist in weight management.	• Significant decrease in mean weight and waist circumference from baseline to post-intervention (week 6) and follow-up (week 12; 72.2 ± 10.4, 71.6 ± 10.8, 71.4 ± 11.0 kg, *p*=0.008, and 92.1 ± 10.1, 89.9 ± 9.9, 87.8 ± 10.7 cm, *p*<0.001, respectively).<br>• Significant increase in self-efficacy for healthy eating and physical activity, eating behaviours, and	• The app-based weight management program was useful for the prevention and control of overweight and obesity.

		dietary intake pattern of the participants (*p*=0.002, *p* <0.001, and *p*<0.001), respectively.	
Lin, 2018 [47]	To monitor vital signs.	• The accuracy rate of up to 96% for predicting abnormality of cardiac functions.	• TAM responses found that eight out of ten response criteria supported favourably to the use of wearable sensors.<br>• Smart clothing was capable of predicting the abnormality of cardiac functions.
Prada, 2017 [48]	To monitor & regulate stress.	• Decrease in the average aversive tension measured (Initial: M = 5.95 (SD=3.13) points, Final: M = 2.83 (SD=2.36) points, (paired t-test = 3.18; *p*<0.05)).<br>The app was mainly used during the day between 10 and 11 a.m. and between 8 and 10 p.m.	• App-based interventions assisted in reducing aversion tension and was user-friendly.

QLQ-C30: Quality of Life Questionnaire-Core 30; EDE-Q: Eating Disorder Examination Questionnaire; TAM: Technology Acceptance Model.
